# Reassessment of the electrical connection between the pulmonary veins and the left atrium: A study to determine the different contributions of myocardial fibers along the standard ablation circumference

**DOI:** 10.3389/fcvm.2023.1162197

**Published:** 2023-06-06

**Authors:** Bieito Campos-García, Concepción Alonso-Martín, José M. Guerra, Zoraida Moreno-Weidmann, Francisco Méndez-Zurita, Rodolfo Montiel-Quintero, Andrés Betancur-Gutiérrez, Xavier Viñolas-Prat, Enrique Rodríguez-Font

**Affiliations:** Arrhythmia Unit, Department of Cardiology, Hospital de la Santa Creu I Sant Pau, CIBERCV, Institut de Recerca HSCSP-IIB Sant Pau, Universitat Autònoma de Barcelona, Barcelona, Spain

**Keywords:** atrial fibrillation, ablation, pulmonary vein, electrical connection, myocardial fiber

## Abstract

**Background:**

Circumferential ablation around the ipsilateral pulmonary veins (PVs) is the standard strategy for atrial fibrillation ablation. The present study seeks to assess which regions of the standard ablation circumference are the main contributors to the venoatrial electrical connection.

**Methods:**

A total of 41 patients were included under a specific atrial fibrillation ablation protocol in which the anterior and posterior segments of the standard circumference, between the equatorial line of the superior and the inferior ipsilateral PVs, were ablated first. If PV isolation was not achieved, ablation was extended superiorly or inferiorly, on the basis of the earliest atrial activation recorded during pacing from inside the PV. Complete PV isolation and the length of the areas not requiring ablation (ANRA) at the time of electrical isolation were evaluated.

**Results:**

Ablation of the anterior and posterior segments of the standard circumference led to the isolation of 77% left-PV pairs and 51% right-PV pairs (*p* = 0,015). A superior extension was required in 23% left-PV pairs and in 46% right-PV pairs, while an inferior extension was required only in 10% left-PV pairs and in 11% right-PV pairs. PV isolation was achieved before completing the standard ablation circumference in 97% left-PV pairs and in 94% right-PV pairs, with a median ANRA of 36.9 (IQR: 30.9–42.1) mm in the left PVs [16.0 (IQR: 12.0–19.0) mm superior and 18.8 (IQR: 16.1–24.9) mm inferior, *p* < 0.01] and 36.9 (IQR: 30.2–41.0) mm in the right PVs [15.1 (IQR: 10.7–19.1) mm superior and 20.6 (IQR: 16.9–23.3) mm inferior, *p* < 0.01].

**Conclusions:**

The myocardial fibers along the anterior and posterior regions of the standard ablation circumference are the main contributors to the electrical connection between the pulmonary veins and the left atrium. Ablation of these regions results in PV isolation in the majority of patients.

## Introduction

**Figure 1:**
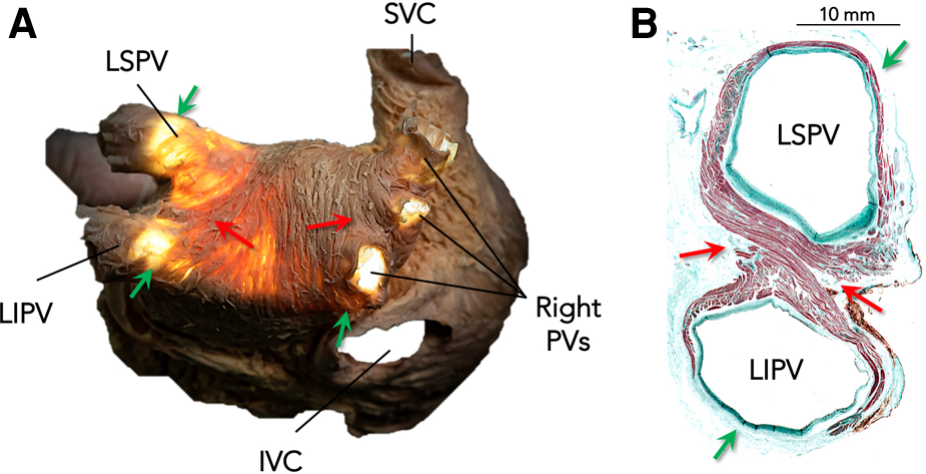
Arrangement of myocardial fibers along the venoatrial junction in the left atrium. (**A**) Dissection showing a posterior view of the left atrium with transillumination to demonstrate the thinner sections. (**B**) A histological section in the transverse plane of a left-PV pair stained with Masson's trichrome technique to show variations in the widths of muscular fibers along the venoatrial junction. Note that myocardial fibers entering the carina are thicker (red arrows), while they are thinner and exhibit discontinuities in the superior and inferior aspects of the PV pairs (green arrows), especially on the left side. LSPV, left superior pulmonary vein; LIPV, left inferior pulmonary vein; PVs, pulmonary veins; SVC, superior vena cava; IVC, inferior vena cava. Histological images provided courtesy of Dr. José Angel Cabrera.

Circumferential ablation around the ipsilateral pulmonary veins (PVs) has been adopted as the standard strategy for PV isolation (1). However, the arrangement of muscle fibers along the junction between the PVs and the left atrium (LA) is complex and heterogeneous. This heterogeneity could result in different contributions of these fibers to the PV–LA electrical connection along the standard ablation circumference (2–4). Previous anatomical and electrophysiological studies have shown that the myocardial fibers located in the carina between the ipsilateral PVs are thicker, markedly intermingled, and critical in maintaining the electrical connection between the LA and the PVs (Figure 1) (5–8).

The present study sought to assess which regions of the standard ablation circumference are the main contributors to the PV–LA electrical connection. Since ablation of the anterior and posterior regions of the standard ablation circumference targets the myocardial fibers entering the carina region, we hypothesized that ablation of these segments could result in the isolation of the ipsilateral PVs before the completion of the circumferential ablation process.

## Materials and methods

### Study population

A total of 41 consecutive patients undergoing a first procedure of radiofrequency (RF) catheter ablation for PV isolation were included. All procedures were performed following the guidelines of the Institutional Committee on Human Research at Hospital Sant Pau, and all patients provided written informed consent.

All procedures were performed under the condition of general anesthesia. Standard multielectrode catheters were advanced inside the coronary sinus and in the His bundle region. A single atrial transseptal puncture was performed using the standard Brockenbrough technique, and a cool-tip ablation catheter and a multielectrode mapping catheter were introduced in the LA. Intracardiac electrograms were recorded using the Prucka CardioLab electrophysiology system (GE Medical Systems, Milwaukee, WI, USA). All patients were in the state of sinus rhythm during mapping, ablation, and evaluation of PV isolation. Electrical cardioversion was performed whenever necessary to revert any episode of atrial fibrillation during the procedure.

High-density electroanatomic maps were created during atrial pacing from the distal coronary sinus using the CARTO 3 system (Carto System®, Biosense-Webster Inc., Diamond Bar, CA, USA) with the PentaRay® 20-pole steerable mapping catheter (Biosense-Webster, Inc., Diamond Bar, CA, USA). The resulting electroanatomical maps were merged with the left atrium images previously obtained by MRI or CT scan using the CartoMerge tool (CartoMerge®, Biosense-Webster, Inc.). Patients with significant areas of bipolar low voltage shown on the high-density map of the left atrium, consistent with scarring (≥1 cm^2^, <0.5 mV), were not included in the study.

Ablation was performed using the irrigated tip contact force–sensing RF ablation catheter Thermocool SmartTouch® (Biosense-Webster Inc., Diamond Bar, CA, USA) with a power of 30 W and a maximum temperature of 43° at a flow rate of 20 ml/min. The target ablation index was 420 in the anterior wall and 370 in the posterior wall with an interlesion distance ≤5 mm.

### Ablation protocol

**Figure 2:**
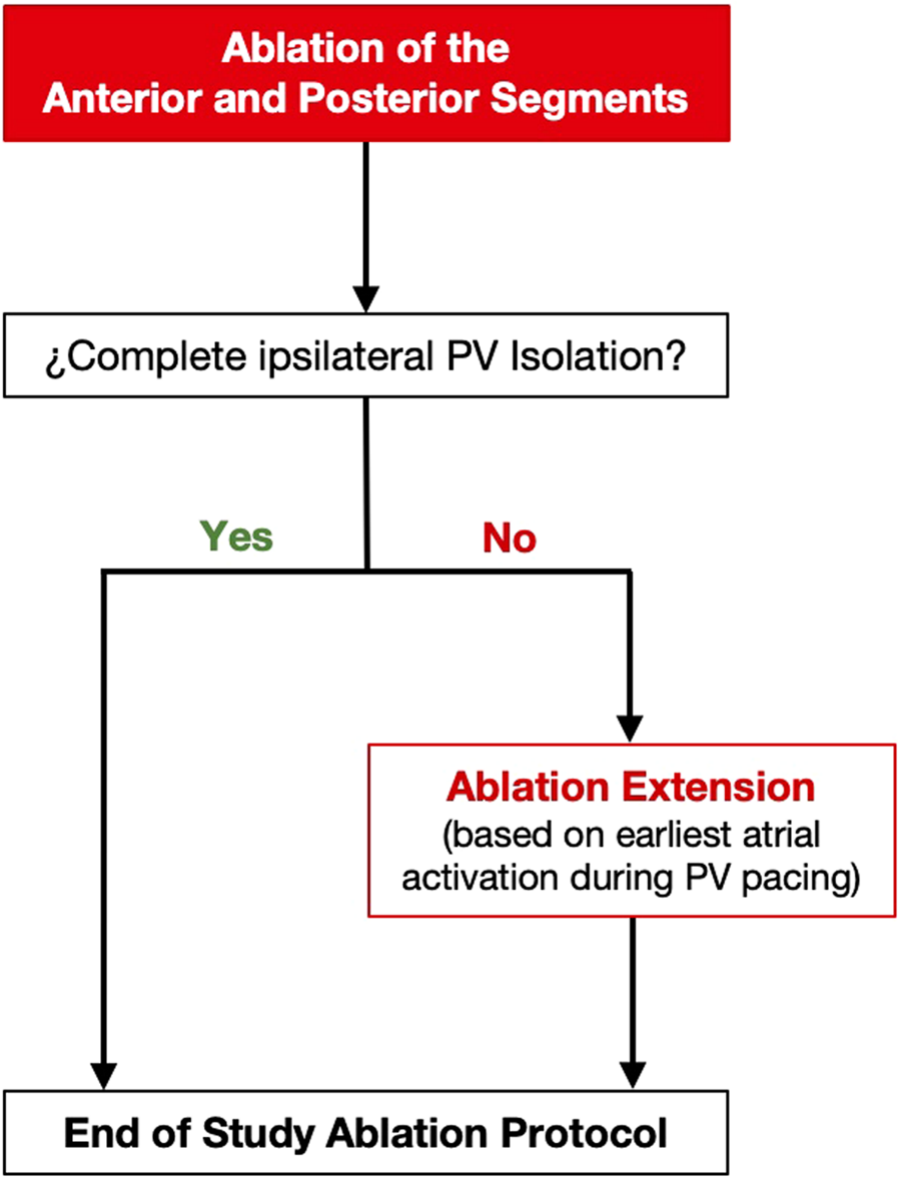
Flowchart of the study ablation protocol.

**Figure 3:**
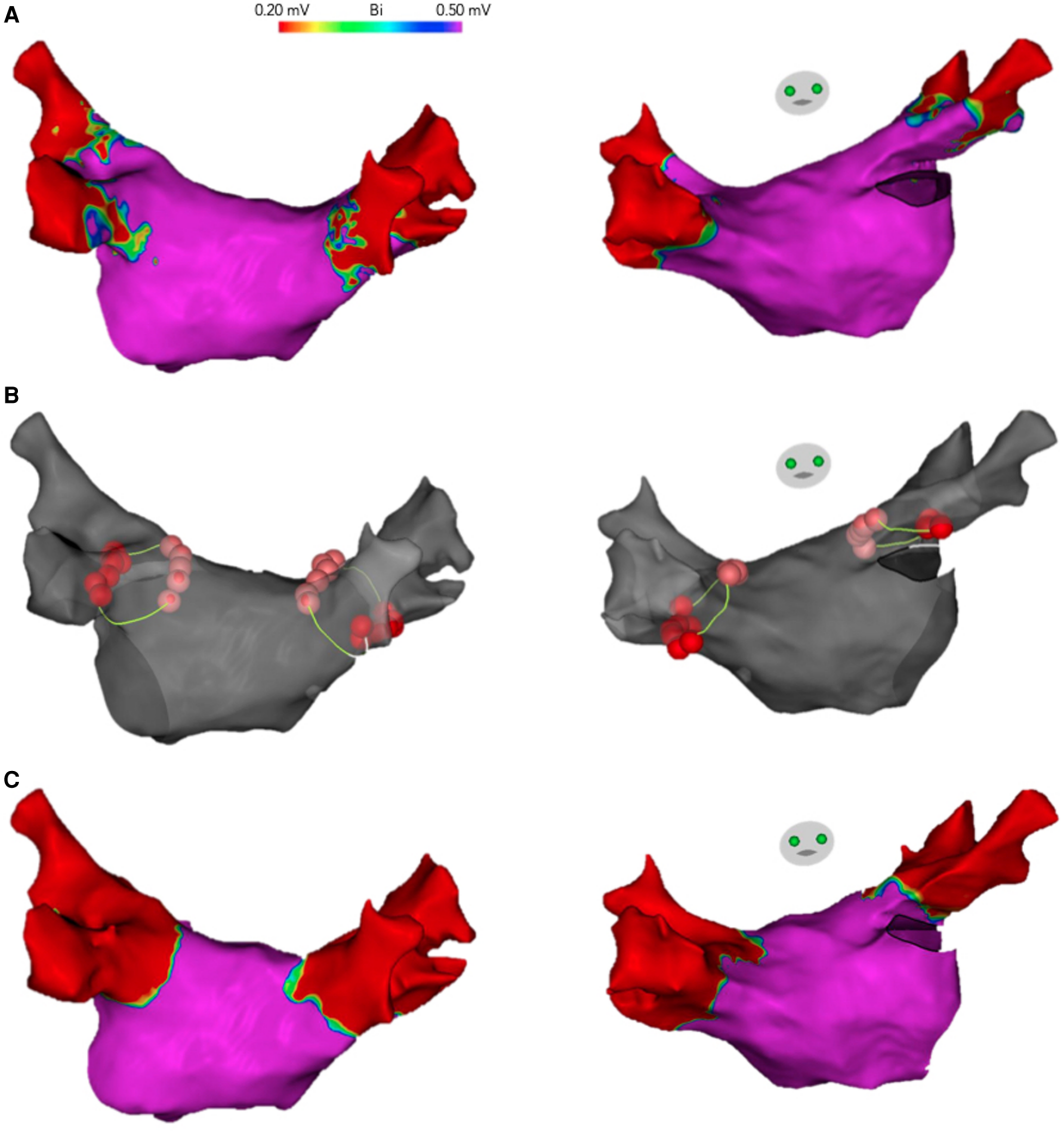
Complete isolation of left and right-PV pairs after ablation of the anterior and posterior segments of the standard circumference around the ipsilateral PVs. Posterior and anterior views of the LA electroanatomical maps are shown. (**A**) High-density voltage map of the LA obtained before ablation. (**B**) The electroanatomical map is shown in gray with the RF lesions (ablation tags), delivered only in the anterior and posterior segments of the standard circumference around the ipsilateral PVs (yellow line), resulting in complete electrical isolation. (**C**) High-density voltage map acquired after PV isolation. Note that the low-voltage areas, consistent with electrical isolation, extend all around the ipsilateral PVs, including the upper and lower aspects of the PV pair, where ablation was not performed (ANRA).

**Figure 4:**
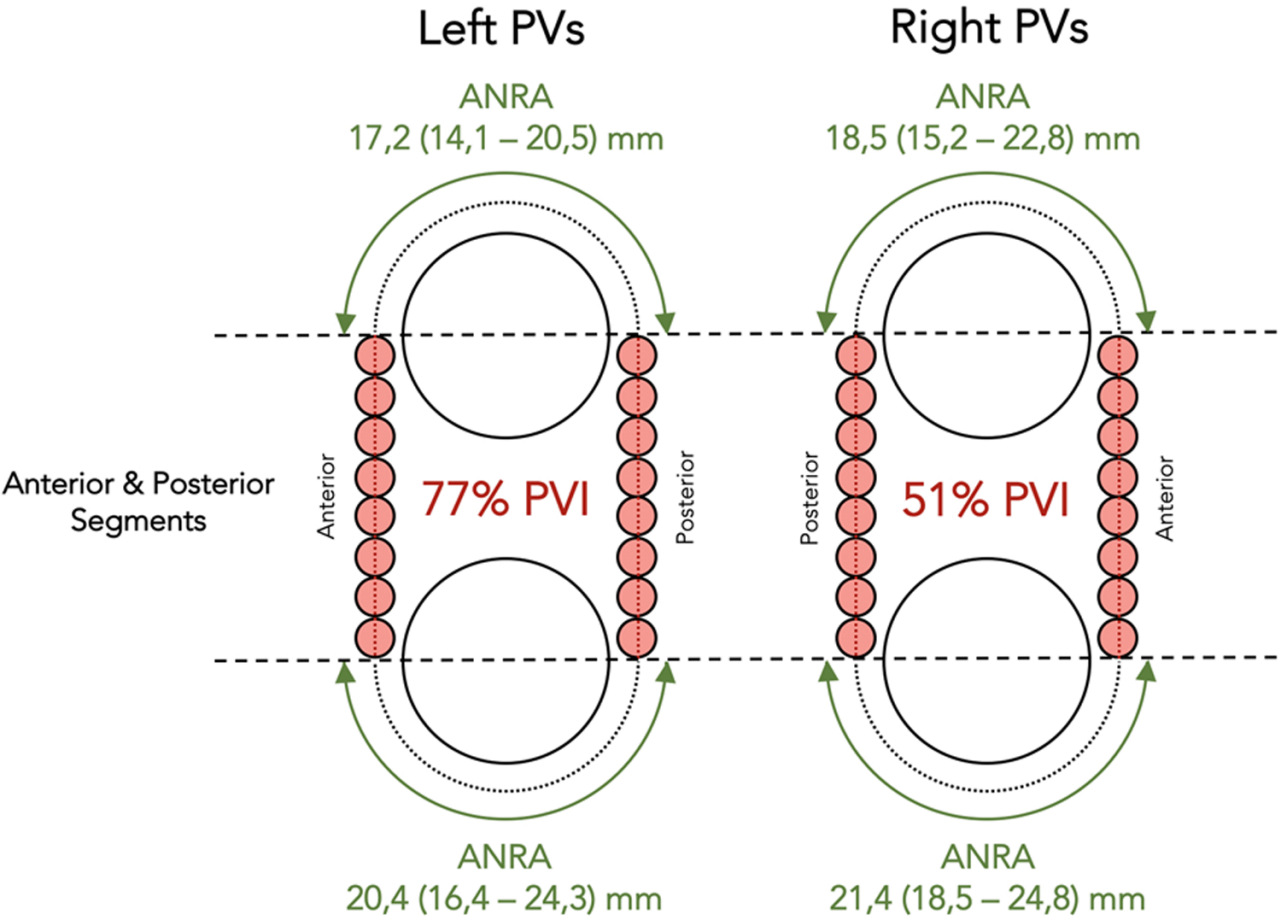
Schematic representation illustrating the observations after ablation to the anterior and posterior segments of the standard circumference around the ipsilateral PVs. Ablation in these segments (orange dots) resulted in complete PV isolation of 77% left-PV pairs and 51% right-PV pairs. Horizontal dashed lines have been drawn to mark the equatorial level of the PVs. The green double-headed arrows show the extent of the areas non-requiring ablation at the time of PV isolation. ANRA, areas not requiring ablation; PVs, pulmonary veins; PVI, pulmonary vein isolation. ANRA length is expressed as median (IQR).

We designed an ablation protocol in which the anterior and posterior segments of the standard circumference around the ipsilateral veins were ablated first (Figure 2). The length of these segments was empirically defined from the equatorial level of the superior PV to the equatorial level of the inferior PV (Figures 3, 4). Ablation was initiated on the left side PVs with the PentaRay catheter placed in the superior PV. Radiofrequency applications were delivered along the anterior segment in a downward direction, and along the posterior segment, in an upward direction. Once ablation at both the anterior and the posterior segments was performed, PV electrical connection was evaluated with the PentaRay catheter. Pacing from inside the PVs was systematically performed by using the PentaRay catheter to assess the exit block and confirm PV isolation. In the event that both ipsilateral PVs were isolated, a new high-density voltage map of the PV pair and the adjacent LA was acquired to further confirm complete PV isolation and to assess the extent of voltage changes relative to the baseline voltage map (Figure 3).

**Figure 5:**
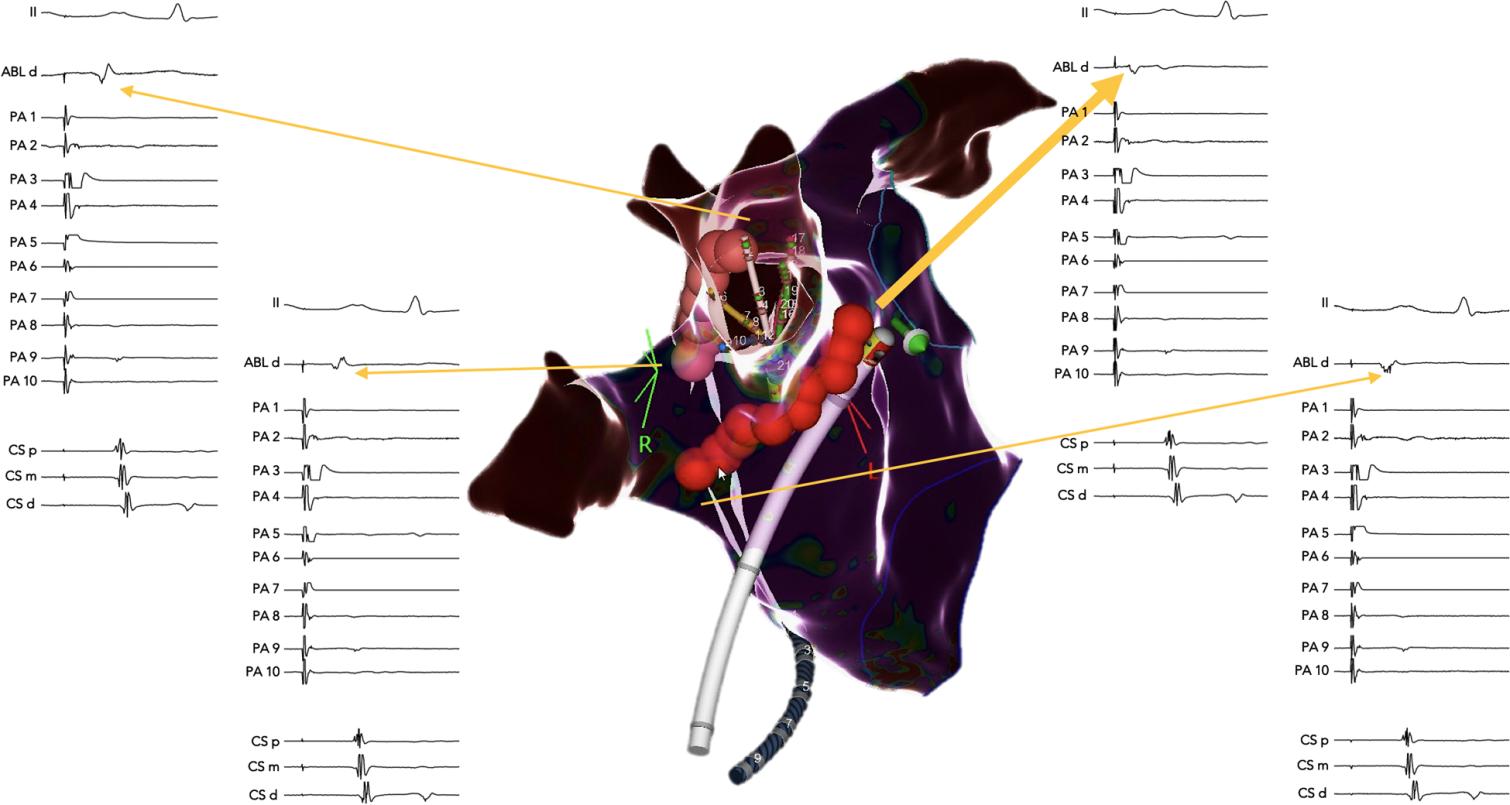
Example of ablation extension from the anterior and posterior segments guided by pacing from inside the connected PVs. A right lateral view of the right PVs that remained connected after ablation to the anterior and posterior segments of the standard circumference. The pacing was performed from the multipolar catheter inside the right-superior PV. Atrial activation next to the ablated segments was mapped using the ablation catheter. Note the earliest atrial activation identified at the superior end of the anterior ablated segment (thick yellow arrow), where two additional RF lesions led to complete PV isolation of both the superior and the inferior right PVs.

In the case that any of the ipsilateral PVs remained connected, atrial activation adjacent to the ablated segments was mapped using the ablation catheter, during pacing from inside the PV with the PentaRay® catheter. Based on the earliest atrial activation, one of the ablated segments was extended either upward, around the superior PV, or downward, around the inferior PV, until PV electrical isolation was achieved (Figures 2, 5).

**Figure 6:**
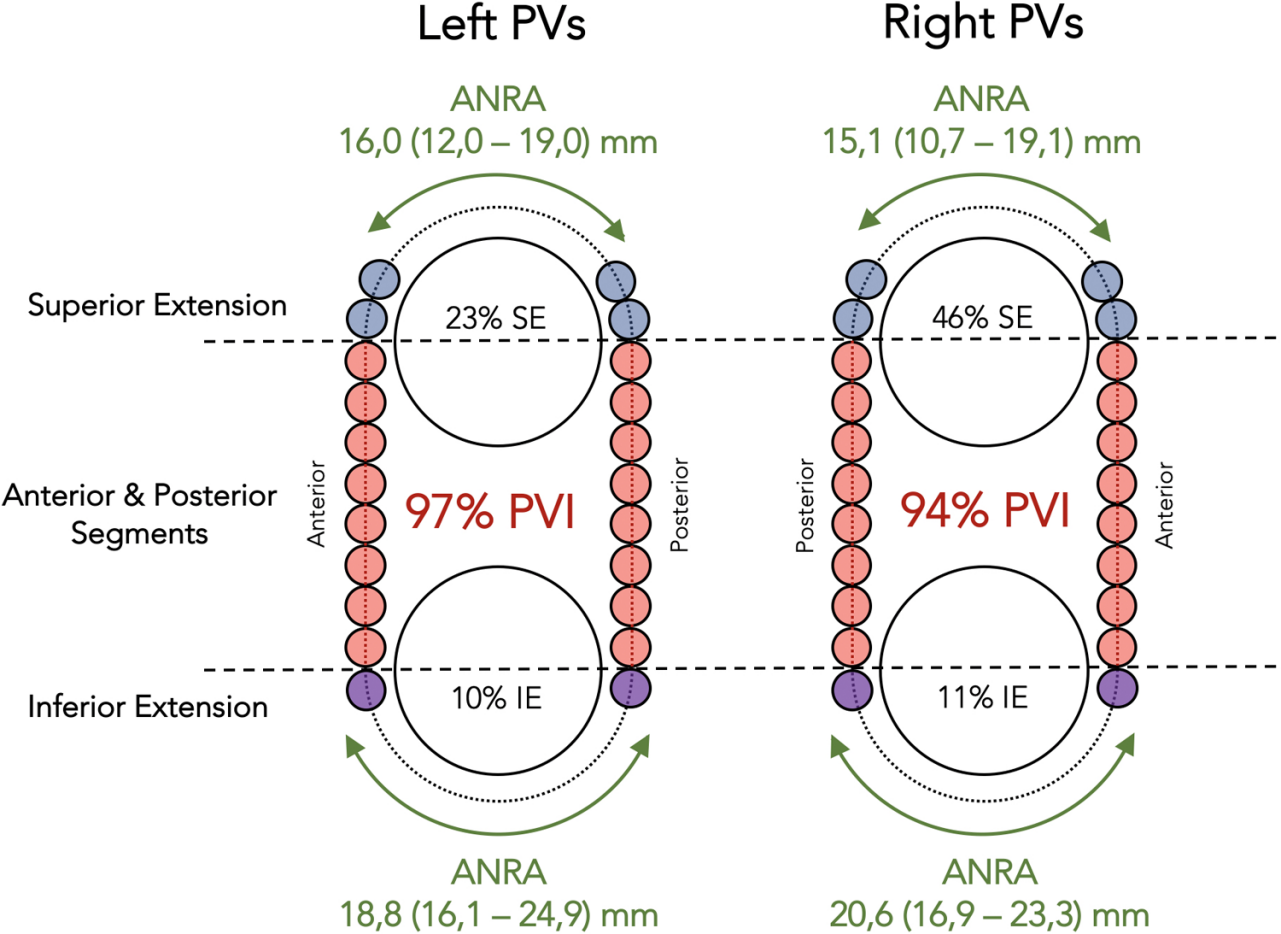
Schematic representation illustrating the observations at the end of the ablation protocol. As in Figure 4, horizontal dashed lines have been drawn to mark the equatorial level of the PVs, and the orange dots represent the initial set of RF lesions to the anterior and posterior segments of the standard circumference around the ipsilateral PVs. The blue and purple dots represent additional RF lesions in those patients requiring ablation extension from the anterior and posterior segments. The green double-headed arrows show the extent of the areas not requiring ablation at the time of PV isolation. ANRA, areas not requiring ablation; PVs, pulmonary veins; PVI, pulmonary vein isolation; SE, superior extension; IE, inferior extension. ANRA length is expressed as median (IQR).

The rate of complete PV isolation before closing the standard ablation circumference and the length of the areas not requiring ablation (ANRA) at the time of PV isolation were evaluated (Figures 4, 6).

Regardless of whether PV isolation was achieved at the end of the ablation protocol, completion of the circumferential standard ablation process was always achieved around the ipsilateral PVs. First-pass ablation was considered when PV isolation was obtained during the implementation of the ablation protocol or at the completion of the circumferential line. PV groups without first-pass isolation were excluded from the analysis.

### Statistical analysis

Continuous data are expressed as mean ± standard deviation or median (interquartile range) when appropriate. Categorical variables are expressed in percentages. All continuous data were tested using the one-sample Kolmogorov–Smirnov test against a normal distribution. A comparison between groups was made using the Student’s t-test, the Mann–Whitney *U*-test, and the *X*^2^ test, according to the type of variable and its distribution. A *p*-value <0.05 was considered statistically significant. The analysis was conducted by using the IBM SPSS Statistics version 26.0 (SPSS, Chicago, IL, USA).

## Results

### Patient characteristics

The baseline characteristics of the 41 patients in the study are listed in Table 1. A total of 29 (71%) patients had paroxysmal AF, and 12 (29%) had persistent AF. Four patients (10%) had known structural heart disease: two with ischemic heart disease with preserved left ventricular systolic function and two with non-ischemic disease with mild and moderately reduced left ventricular ejection fraction due to tachycardiomyopathy, and these conditions normalized after good rhythm control was achieved in both patients. On preprocedural imaging assessment, 20 patients (49%) were found to have a left common ostium, one patient (2%) had a right common ostium, and seven (17%) had an additional right PV.

**Table 1:** Baseline characteristics of the patients included in the study.

*N*	41
Age (years)	59 (53–64)
Male (%)	30 (73)
Hypertension (%)	15 (37)
Diabetes mellitus (%)	3 (1)
Structural heart disease (%)	4 (10)
LVEF	0.60 (0.56–0.60)
Left atrium diameter (mm)	42 (39–46)
Type of atrial fibrillation (%)	
Paroxysmal	29 (71)
Persistent	12 (29)
Antiarrhythmic drugs (%)	36 (88)
Flecainide	18 (44)
Amiodarone	17 (41)
Dronedarone	1 (2)
LA anatomy (%)	
Left common trunk	20 (49)
Right common trunk	1 (2)

Data are expressed as median (IQR).

LA, left atrium, LVEF, left ventricular ejection fraction.

Vein potentials were recorded in all PVs prior to ablation. First-pass PV isolation was observed in 39 (95%) left-PV pairs and in 35 (85%) right-PV pairs, which were available for analysis. Procedural data are presented in Tables 2 and 3.

**Table 2:** Procedural data of ablation in left PVs.

Patient #	First pass PVI	Complete PVI after ablation to anterior/posterior segments	Extension of anterior/posterior ablation needed	Superior ANRA length (mm)	Inferior ANRA length (mm)
1	Yes	Yes	No	12.9	18.7
2	Yes	Yes	No	24.7	23.8
3	Yes	Yes	No	19.0	18.0
4	Yes	Yes	No	18.0	15.3
5	Yes	Yes	No	18.9	23.0
6	Yes	Yes	No	18.0	24.1
7	Yes	Yes	No	24.1	27.3
8	Yes	Yes	No	24.9	18.5
9	Yes	No	Superior	8.7	26.4
10	Yes	Yes	No	12.0	13.3
11	Yes	Yes	No	22.9	17.8
12	Yes	No	Superior + inferior	9.8	6.7
13	Yes	No	Superior + inferior	0	0
14	Yes	Yes	No	22.3	25.0
15	Yes	No	Superior + inferior	5.6	12.0
16	Yes	Yes	No	12.2	25.9
17	Yes	No	Superior	12.0	18.8
18	Yes	Yes	No	14.3	22.7
19	Yes	Yes	No	16.4	23.4
20	Yes	Yes	No	18.3	18.5
21	Yes	Yes	No	14.0	8.0
22	Yes	No	Superior + inferior	11.9	14.6
23	Yes	Yes	No	22.6	25.1
24	Yes	Yes	No	14.7	22.1
25	Yes	Yes	No	17.3	17.1
26	Yes	Yes	No	15.7	24.4
27	No	N/A	N/A	N/A	N/A
28	Yes	Yes	No	26.6	25.2
29	Yes	Yes	No	9.0	16.2
30	Yes	Yes	No	20.7	24.0
31	Yes	Yes	No	17.0	26.5
32	Yes	No	Superior	9.0	25.0
33	Yes	Yes	No	20.0	17.0
34	No	N/A	N/A	N/A	N/A
35	Yes	No	Superior	12.0	26.1
36	Yes	Yes	No	14.0	12.0
37	Yes	Yes	No	15.9	15.3
38	Yes	Yes	No	16.1	26.7
39	Yes	No	Superior	18.6	18.0
40	Yes	Yes	No	10.5	10.9
41	Yes	Yes	No	11.5	16.0

ANRA, areas not requiring ablation; PVI, pulmonary vein isolation; N/A, not applicable.

**Table 3:** Procedural data of ablation in right PVs.

Patient #	First pass PVI	Complete PVI after ablation to anterior/posterior segments	Extension of anterior/posterior ablation needed	Superior ANRA length (mm)	Inferior ANRA length (mm)
1	Yes	Yes	No	25.3	26.3
2	Yes	Yes	No	23.2	29.3
3	No	N/A	N/A	N/A	N/A
4	Yes	No	Superior	16.9	23.3
5	Yes	No	Superior	15.2	33.5
6	Yes	Yes	No	21.8	24.6
7	Yes	No	Superior	10.8	21.2
8	Yes	Yes	No	23.1	28.6
9	Yes	No	Superior + inferior	12.9	20.3
10	Yes	Yes	No	20.8	29.3
11	No	N/A	N/A	N/A	N/A
12	Yes	No	Superior	6.2	28.9
13	Yes	No	Superior + inferior	0	0
14	Yes	No	Superior	12.4	21.9
15	Yes	Yes	No	18.0	20.0
16	Yes	Yes	No	13.3	24.8
17	Yes	No	Superior	13.2	16.3
18	Yes	No	Inferior	16.0	0
19	Yes	Yes	No	19.1	18.0
20	Yes	Yes	No	10.1	14.3
21	No	N/A	N/A	N/A	N/A
22	Yes	Yes	No	19.0	22.0
23	Yes	No	Superior	9.5	23.0
24	Yes	Yes	No	9.0	12.7
25	Yes	Yes	No	11.8	23.0
26	Yes	Yes	No	15.9	21.7
27	Yes	No	Superior	4.8	14.5
28	Yes	No	Superior	10.0	20.0
29	Yes	No	Superior + inferior	6.0	13.0
30	Yes	Yes	No	25.0	16.9
31	Yes	No	Superior	20.9	16.3
32	No	N/A	N/A	N/A	N/A
33	Yes	No	Superior	13.9	14.5
34	Yes	No	Superior	10.6	19.6
35	Yes	No	Superior	10.0	14.0
36	No	N/A	N/A	N/A	N/A
37	Yes	Yes	No	16.9	20.0
38	No	N/A	N/A	N/A	N/A
39	Yes	Yes	No	15.0	21.0
40	Yes	Yes	No	16.6	20.6
41	Yes	Yes	No	24.0	17.0

ANRA, areas not requiring ablation; PVI, pulmonary vein isolation; N/A, not applicable.

### Pulmonary vein isolation during ablation to the anterior and posterior segments of the standard circumference

Ablation of the anterior and posterior segments of the standard circumference resulted in complete electrical isolation of both ipsilateral veins in 30 (77%) left-PV pairs and in 18 (51%) right-PV pairs (*p* = 0.015) (Figures 3, 4).

In the left PVs, ablation of the anterior segment required a median of 9 (IQR: 7–10) RF lesions, while the posterior segment required 7 (IQR: 6–8) RF lesions. The number of RF lesions required to achieve PV isolation was significantly lower than the total lesions needed to complete the ablation of the circumference in these PV pairs, 17 (IQR: 12–19) vs. 26 (IQR: 21–28) (*p* < 0.01). The median length of the ANRA at the time of complete PV isolation was 17.2 (IQR: 14.1–20.5) mm in the superior aspect of the circumference and 20.4 (IQR: 16.4–24.3) mm in the inferior aspect of the left circumference.

In the right PVs, ablation of the anterior segment required a median of 9 (IQR: 8–10) RF lesions, while ablation of the posterior segment required 8 (IQR: 7–10) RF lesions. The number of RF lesions required to achieve PV isolation was also lower than the total lesions needed to complete the ablation of the circumference in these PV pairs, 17 (IQR: 15–20) vs. 27 (IQR: 24–29) (*p* < 0.01). The median length of the ANRA at the time of complete PV isolation was 18.5 (IQR: 15.2–22.8) mm in the superior aspect and 21.4 (IQR: 18.5–24.8) mm in the inferior aspect of the right circumference.

### Extended ablation from the initial anterior and posterior segments

Nine left-PV pairs (23%; 9 left-superior and 8 left-inferior PVs) and 17 right-PV pairs (49%; 16 right-superior and 12 right-inferior PVs) remained connected after ablation of the anterior and posterior segments. In these cases, the initially ablated segments were extended on the basis of the earliest atrial activation identified during pacing from inside the connected PV (Figure 5).

#### Left pulmonary veins

A superior extension from the initially ablated segments was required in all nine left-PV pairs that remained connected, five anteriorly and four posteriorly. The superior ablation extensions achieved the isolation of eight left-superior PVs and of four left-inferior PVs after a median of three (IQR: 2–4) additional RF lesions. The left-superior PV that remained connected was successfully isolated during the subsequent inferior ablation extension.

An inferior extension from the initially ablated segments was required in four of the nine left-PV pairs that remained connected, two anteriorly and two posteriorly. These inferior ablation extensions achieved the isolation of four left-inferior PVs and one left-superior PV after a median of four (IQR: 3–4) additional RF lesions.

The number of RF lesions required to achieve PV isolation after ablation extension was still significantly lower than the total lesions needed to complete the ablation of the circumference in these PV pairs, 18 (IQR: 15–25) vs. 25 (IQR: 24–29) (*p* = 0.028).

#### Right pulmonary veins

A superior extension from the initially ablated segments was required in 16 of the 17 right-PV pairs that remained connected, 14 anteriorly and two posteriorly. The superior ablation extensions achieved the isolation of 15 right-superior PVs and of eight right-inferior PVs after a median of two (IQR: 2–3) additional RF lesions. The right-superior PV that remained connected was successfully isolated during the subsequent inferior ablation extension.

An inferior extension from the initially ablated segments was required in four of the 17 right-PV pairs that remained connected, two anteriorly and two posteriorly. These inferior ablation extensions achieved the isolation of four right-inferior PVs and one right-superior PV after a median of two (IQR: 1–2) additional RF lesions.

The number of RF lesions required to achieve PV isolation after ablation extension was also significantly lower than the total lesions needed to complete the ablation of the circumference in these PV pairs, 21 (IQR: 19–22) vs. 27 (IQR: 26–29) (*p* < 0.01).

#### Completion of ablation protocol

At the end of the ablation protocol, complete PV isolation was achieved before the standard ablation circumference around the ipsilateral PVs was closed in 38 (97%) left-PV pairs and in 33 (94%) right-PV pairs. The number of RF lesions required to achieve PV isolation was significantly lower than the total lesions needed to close the standard ablation circumference around the ipsilateral PVs: 17 (IQR: 14–19) vs. 25 (IQR: 23–28) in the left PVs and 19 (IQR: 16–21) vs. 27 (IQR: 25–29) in the right PVs (*p* < 0.01). The total length of the ANRA was median 36.9 (IQR: 30.9–42.1) mm in the left PVs [16.0 (IQR: 12.0–19.0) mm superior and 18.8 (IQR: 16.1–24.9) mm inferior, *p* < 0.01] and 36.9 (IQR: 30.2–41.0) mm in the right PVs [15.1 (IQR: 10.7–19.1) mm superior and 20.6 (IQR: 16.9–23.3) mm inferior, *p* < 0.01] (Figure 6).

## Discussion

The main finding of our study is that ablation of the anterior and posterior regions of the standard ablation circumference resulted in complete electrical isolation in 97% of left-PV pairs and in 94% of right-PV pairs, leaving large areas that did not require ablation (ANRA). This confirms the hypothesis that the muscle fibers running through these regions are the main contributors to the electrical connection between the LA and the PVs.

The arrangement of atrial myocardial fibers and their contribution to the PV–LA electrical connection is not considered during the circumferential ablation of atrial fibrillation. However, we know from anatomical studies that this arrangement is heterogeneous and complex, with myocardial fibers that are thicker and markedly intermingled in the venoatrial junction near the carina between the ipsilateral PVs, whereas they are thinner or may be even absent in other venoatrial junction regions (Figure 1) (2–5).

Our study demonstrates the critical role of the muscular fibers running through the anterior and posterior segments of the standard ablation circumference for the PV–LA electrical connection, as the initial ablation set, targeting only these segments, was able to isolate up to 77% of the left and 51% of the right ipsilateral PVs. Interestingly, most of the PV pairs that remained connected were isolated with minimal ablation extensions, mainly directed to the superior veins. Therefore, the largest ANRAs were observed at the inferior aspect of both left and right-PV pairs. This finding is consistent with anatomical studies showing that muscular sleeves are more extensive around the superior than around the inferior PVs and that the inferior aspect of the inferior PVs is generally devoid of muscular fibers (2).

In Figure 3 we illustrate how, after ablation of only the anterior and posterior segments of the standard circumference, the low voltage area extends all around the ipsilateral PVs, including the upper and lower aspects of the PV pair, where ablation was not performed (ANRA). Some anatomical features may help us to understand the complete isolation of the ipsilateral pulmonary veins despite leaving large areas unablated. First, the presence of longitudinal fascicles along the venoatrial junction with the potential for myocardial continuity between adjacent veins may explain our observation that minimal superior ablation extensions led to the isolation of several inferior PVs and that some inferior ablation extensions also led to the isolation of some superior PVs. Second, myocardial discontinuities related to acquired areas of fibrosis have been described in the myocardial sleeves that enter the PVs. Third, the ostium of the PVs is surrounded by circular fiber bundles that are arranged transversally to the vein axis. We hypothesize that RF lesions delivered to the anterior and posterior aspects of the ablation circumference could damage these fibers and produce conduction blocks along them (2–5, 9).

Our study aims to deepen the knowledge of the electrical connection between the PVs and the LA by using an anatomical approach. Our data support the major role of the anterior and posterior regions of the standard ablation circumference for the venoatrial electrical connection. This is particularly relevant at the present time, when the standard approach for PV isolation is being redefined in a new era ushered in by single-shot ablation techniques, such as the recently emerged pulsed field ablation, which is clinically promising but still has non–well-established protocols of energy delivery along the venoatrial junction. We hope that the information provided by our study will be clinically useful and may allow optimization of the standard approach for PV isolation.

## Study limitations

The number of patients included in the study is relatively small and reflects their experiences in a single center.

The clinical effect of the proposed ablation strategy cannot be evaluated, since the entire standard circumferential ablation line was systematically completed around the ipsilateral PVs, regardless of whether PV isolation had been achieved during the implementation of the ablation protocol. However, the objective of this study was not to evaluate the clinical impact of this ablation strategy but to determine the regional contribution to the PV–LA electrical connection along the standard ablation circumference. Our results represent a proof of concept that complete PV isolation may be achieved before the standard ablation of the circumference is completed, leaving large areas with no ablation at the time of PV isolation. The clinical effect of a PV isolation strategy with ablation restricted to the anterior and posterior regions of the standard circumference around the ipsilateral PVs remains unknown and warrants further study.

## Conclusion

The myocardial fibers along the anterior and posterior regions of the standard ablation circumference are the main contributors to the electrical connection between the pulmonary veins and the left atrium. Ablation of these regions results in PV isolation in the majority of patients.

## Data Availability

The original contributions presented in the study are included in the article, and further inquiries can be directed to the corresponding author.
